# Emotion regulation predicts pain and functioning in children with juvenile idiopathic arthritis

**DOI:** 10.1186/1546-0096-10-S1-A32

**Published:** 2012-07-13

**Authors:** Mark Connelly, Kelly K Anthony, Maggie H Bromberg, Lindsey Franks, Karen M Gil, Laura E Schanberg

**Affiliations:** 1 Children's Mercy Hospitals and Clinics, Kansas City, MO, USA; 2 Duke University Medical Center, Durham, NC, USA; 3 University of North Carolina at Chapel Hill, Chapel Hill, NC, USA

## Purpose

Understanding variables that predict increased pain and disability in children with JIA remains an important avenue for research and intervention. The present study sought to evaluate the extent to which regulation of positive and negative emotions predicts pain intensity and disability at a given moment for children with JIA.

## Methods

The study sample included 43 children (37 female) ages 8-17 years (M = 12.77 years, SD = 2.29) previously diagnosed with Juvenile Idiopathic Arthritis (JIA) and having active arthritis in the past 6 months. During an initial study visit, participants were trained in the use of a Smartphone for answering questions about pain, activity limitations, emotions, and use of emotion regulation strategies. Participants then were cued via audible alerts to respond to these questions three times per day for 4 weeks. Hierarchical linear models were used to evaluate the following: (a) whether pain and functional limitations were reliably greater for more emotionally labile children (i.e., children with high variability in positive and negative emotions); (b) whether pain and functional limitations reliably changed during moments when positive and negative emotions were higher or lower than typical for a given child; and (c) whether pain and functional limitations reliably changed during intervals when positive and negative emotions were “successfully” regulated (i.e., either recovered to or maintained within .5 standard deviations of a child’s typical positive or negative emotion intensity following use of one or more emotion regulation strategies).

## Results

Pain intensity was significantly higher for children with greater negative and positive emotion lability (*b*=55.68, t(41)=2.17, p=.02; *b*=60.29, t(41)=2.18, p=.04) and at moments when negative and positive emotion intensities were higher or lower (respectively) than a given child’s typical level (*b*=3.14, t(2389)=-3.13, p<.01; *b*=-5.49, t(2389)=-8.01, p<.01). Pain intensity was significantly lower during intervals when negative and positive emotions were successfully maintained at adaptive levels (*b*=-2.51, t(1992)=-2.68, *p*<.01; *b*=-5.12, t(1870)=-5.82, *p*<.01) and when positive emotions were recovered to adaptive levels following a significant drop (*b*=-5.14, t(1549)=-4.71, *p*<.01). Limitations in daily activities were reliably greater for children with greater negative emotion lability (*b*=12.93, t(41)=2.09, p=.02) and at moments when negative emotion intensities were lower than a child’s typical level (*b*=1.11, t(2389)=-3.14, p<.01). Activity limitations were significantly lower during intervals when positive emotions were successfully maintained at adaptive levels (*b*=-.62, t(1870)=-2.00, p=.04) or negative emotions were recovered to adaptive levels following a significant rise (*b*=-1.42, t(804)=-2.59, p=.01).

## Conclusion

The extent of overall pain and activity limitations among children with JIA is partly influenced by individual differences in emotion lability and moment-to-moment differences in the intensities and regulation of positive and negative emotions. Interventions that target the ability to adaptively modulate emotions may help reduce pain and improve quality of life for children with JIA.

## Disclosure

Mark Connelly: None; Kelly K. Anthony: None; Maggie H. Bromberg: None; Lindsey Franks: None; Karen M. Gil: None; Laura E. Schanberg: None.

